# An IoT-Enabled Information System for Smart Navigation in Museums

**DOI:** 10.3390/s22010312

**Published:** 2021-12-31

**Authors:** Muhammad Nawaz Khan, Haseeb Ur Rahman, Mohammad Faisal, Faheem Khan, Shabir Ahmad

**Affiliations:** 1 Network System & Security Research Group, Department of Computer Science & IT, University of Malakand, Chakdara 18800, Pakistan; nawazpk805@uom.edu.pk (M.N.K.); haseeburrahman@uom.edu.pk (H.U.R.); mfaisal@uom.edu.pk (M.F.); 2 Department of Computer Engineering, Gachon University, Seongnam 13120, Korea; shabir@gachon.ac.kr

**Keywords:** IoT, sensor network, energy, navigation system, pervasive computing

## Abstract

The Internet of Things (IoT) is a new paradigm that connects objects to provide seamless communication and contextual information to anyone, anywhere, at any time (AAA). These Internet-of-Things-enabled automated objects interact with visitors to present a variety of information during museum navigation and exploration. In this article, a smart navigation and information system (SNIS) prototype for museum navigation and exploration is developed, which delivers an interactive and more exciting museum exploration experience based on the visitor’s personal presence. The objects inside a museum share the information that assist and navigate the visitors about the different sections and objects of the museum. The system was deployed inside Chakdara Museum and experimented with 381 users to achieve the results. For results, different users marked the proposed system in terms of parameters such as interesting, reality, ease of use, satisfaction, usefulness, and user friendly. Of these 381 users, 201 marked the system as most interesting, 138 marked most realistic, 121 marked it as easy-in-use, 219 marked it useful, and 210 marked it as user friendly. These statistics prove the efficiency of SNIS and its usefulness in smart cultural heritage, including smart museums, exhibitions and cultural sites.

## 1. Introduction

A museum is a place where cultural heritage (CH) is preserved by collecting and arranging various sorts of historical objects in a systematic order to tell the story of history. Visitors to a museum or a heritage site, on the other hand, often have a limited time to stay there. Discovering items of interest and items of a given culture is important for better understanding CH and efficient use of time. It is particularly difficult for a lay person to understand the layout of culture at a museum, exhibition, or heritage site. It is usually not easy to connect items in a gallery/museum according to some historical background and understand the beauty/history of a culture. Museums also do not have enough resources (guides) to guide and explain each item and everything to all visitors. Different people visit the museum and cultural sites to view and understand an ancient culture, particularly for an expert such as an archaeologist. The physical objects in museums, such as statues, coins, swords, musical instruments, etc., belong to prehistoric and historic periods [1]. These objects are lifeless and dreary, having no understanding of what has been happening in the surroundings [2,3]. These lifeless objects need to be organized and streamlined into a uniform layered. These objects need a description of what was happening in the scene/past.

Coupling the IoT [4,5] wireless network [6,7], mobile ad hoc network [8,9,10,11], and context-aware computing enables modern-day museums to provide more interactive and efficient ways of understanding CH through tangible and intangible objects. The tangible objects include archaeological sites, natural environment, and artifacts. The intangible objects include the voices, oral history, and virtual and augmented reality-based experience of cooking food, clothing styles, religious rituals, etc. By embedding sensing, processing, and communication capabilities, the cultural objects become intelligent and interact with each other using the IoT-enabled technologies [12,13]. The context-aware IoT-based solutions can enhance the learning process and save the time and cost of experts [14,15]. These connected objects use sensors for sensing their surrounding and detect nearby user movements. They collect data about near statues and receive visitor’s requests. All of these data are analyzed by the processor, that then responds to visitors via radio-link and transmits it to the main servers. These lifeless objects become more interactive and intelligent after being embedded with these tiny devices. These objects are now responsive to user input. These technologies allow the uninteresting museum environment to be transformed into intelligent and smart spaces, improving user experience and increasing productivity in terms of user satisfaction. The information about various lifeless objects is displayed to visitors in the smart museum in a way that visitors can explore the museum according to their interests and preferences.

In this paper, an IoT-enabled smart navigation and information system (SNIS) is proposed for the museums. The proposed system builds a smart IoT-based environment where a visitor can obtain pervasive information by using a mobile device (smartphone or tablet). SNIS provides information about objects in stimulating way through which the visitor not only understands the scene but also correlates an item of interest. Furthermore, the system can serve as a guide, allowing visitors to explore objects from a given culture in a logical and sequential order throughout history. The proposed system not only focuses on giving information, but it also incorporates interactive applications to enhance the user experience. For example, the information may be provided in the form of a story, with content of the story being customized to the visitor’s level of understanding. Inside the museum, exhibitions, and cultural sites, SNIS provides an interactive and collaborative experience to the visitors.

By guiding a user through a culture, the information must be meaningful and relevant to that culture, so that the user is presented with the culture rather than merely information. Furthermore, to make it more visitor-centric, the information presented to visitors is personalized based on each visitor’s profile or personal preferences. SNIS uses interactive storytelling technique to present information to the visitor. The interactive storytelling technique is widely used in the digital entertainment industry for storytelling. In this case, these stories have related an event that happened in the past, which is exhibited by the statues showcased in the museum. Since the museum has items related to different periods of history, therefore, a visitor has the choice to choose a specific time or story in history. Once a story is selected by the visitor using a software application installed in his/her smartphone, the user is then navigated toward the specific section of the museum where items are showcased and located. When a visitor approaches a particular item of interest, the relevant information about the item is displayed to him/her on his/her screen in the form of a story. If a story spans through multiple stone statues, then a visitor is navigated to the next item in the story until the story is complete. If a visitor selects a different story, the system will detect his/her location and direct him/her to the next story. The goal of this paper is to place a visitor in a smart museum environment, locate items in the museum, show information about items that the visitor is interested in, and direct the visitor to different sections of the museum in an interactive and fun-based storytelling manner. The following are the key contributions of the proposed SNIS,

For museum navigation and exploration, we have developed a Smart Navigation and Information System (SNIS) prototype, which enables an interactive and more exciting museum exploration experience based on a visitor’s personal presence. The exhibits in a museum exchange information that guides and directs visitors around the museum’s various sections and objects. SNIS is a navigation system that allows lifeless artwork (statues/objects) to be simulated in an interesting and interesting way. The SNIS fully operates on current technology (smartphones/tablets) to navigate an indoor museum without wearing additional equipment. The SNIS Android app organizes and displays items of interest in various order based on user types (archeologist/nonexpert). It arranges the artwork at the museum in a logical order so that visitors can easily determine where to begin, how to proceed, and where to end. The proposed prototype provides the information in an easy-to-read format (text, audio, and video) in a storytelling manner.

## 2. Related Work

In the recent past, considerable research has been done on the development of smart and pervasive environments using IoT-enabled technology. Most of the literature has focused almost exclusively on application development with predefined images and records. Some of them have discussed the use of wearable devices and virtual reality. Nearly all the schemes mainly focused on the presentation of information in any form. Information is presented in text, audio, and video, but no scheme has worked on stimulation or excitement for the visitor. Following are some of the leading schemes proposed for museums and Cultural Heritage (CH).

For the indoor smart museum, Alletto et al. [16] proposed location-aware architecture. The architecture is implemented with the help of wearable devices with image recognition algorithms and location-aware services. Different formulas are used for RSSI and removing blurredness. The main contribution of this scheme is to remove blurriness by using an enhanced image processing algorithm. However, wearable devices need an extra budget and users feel hesitation in using the wearable devices instead of his/her own smartphone. The number of visitors also determined the quality of audio and video services. Another framework for creating smart CH, Chianese et al. [17] proposed three-tiers architecture. First, the sensor layer is deployed locally. Second, the network layer (gateway) is responsible for data transfer between different networks. Third, the application layer (cultural heritage information system (CHIS)) is deployed for middle-ware applications. CHIS is validated by a case study in the exhibition. The exhibition has seven thematic parts with a total of 271 sculptures from the nineteenth and early twentieth century. These thematic parts are named “Beauty or Truth”. They used user response and behavior (average time, average number of objects, and average rating) for monitoring. The proposed scheme was more focused on user behavior and visitors felt hesitation in answering questions asked at the end of the visit.

Single–smart–space (S3) is an intelligent cultural space-based architecture proposed by Chianese et al. [18]. This multipurpose framework uses a sensor board (smart cricket), proximity strategy, and a multiplatform application. The proximity algorithm detects the objects in the museum while the smart cricket (UNIX based) senses the place and plays the role of mediator between visitor and objects. It detects user movements and provides services according to the context. Smart cricket is used in an outdoor scenario over a wireless fidelity (WiFi) network with automatic synchronization of the multimedia contents. Prototype for development of “S3” is an innovative approach, but it needs many resources and many technical issues for a real-time scenario. Based on TAM (technology acceptance model), Haugstvedt et al. [19] developed a mobile augmented reality application and named it “The Historical Tour Guide”. This app provides an augmented reality realm for huge crowded areas. It works in three phases: the user selects a point of interest on the map, selects appropriate photos from the same point, and searches the entire map. The information is provided in an unexciting manner where users depend more on maps and points of interest. The proposed augmented reality app is a standalone app, having no client–server architecture and it degrades QoS in rich CH environments.

“Talking Museum” is a project proposed for smart CH by Chianese et al. [20]. Bluetooth-enabled technology is deployed for searching to detect the location of user devices. The proposed model is composed of 1) WSNs, in which sensors are positioned on the objects or attached very near these objects; 2) “Gateway Server” accumulates information from the sensors and base station; 3) “Multimedia Content Server” provides multimedia content on requesting the gateway server according to user choices; and 4) “Multimedia Guide App” is responsible for permitting the multimedia contents to the end-user according to his/her choices. Different levels of complexity are defined. “Talking Museum” is an appealing concept for the CH environment, but the implementation of such a multitier system needs many resources. An IoT-based indoor smart museum was proposed by Chianese et al. [21]. The system monitors user movements inside the smart space through sensors that detect user position to specific objects and information present in an audio form. Broadcasting vocal messages from statues creates a real-time talking object in the museum. The data are provided in voice and multimedia forms. The entire IoT three-layered architecture is named CHIS (cultural heritage information system) server. This article is part of a project done at DATABENC laboratory for a real prototype that needs many resources and middle-ware.

A novel multimedia-based smart environment has been proposed by Angelaccio et al. [22]. It uses NFC (near field communication) technology for obtaining and providing multimedia content for users with links and suggestions. The NFC devices obtain data from other devices known as “passive token”. These passive tokens exchange information with NFC devices without physical contact. The case study was conducted with four practical and simple steps within the “SMART ROOM” app. The system was analyzed for the “SMART VILLA” app with a dynamic image gallery. The scheme was mainly based on NFC technology, but this technology did not work if devices were more than 4 m apart. For smart museum and exhibitions, Chianese et al. [23] proposed a framework in a domain expert system that provides information about objects inside the museum and suggests directions to the end-user during a visit. User experiences and domain expert systems are combined for the development of information, resulting in an information booklet. The booklet consists of a two-tier content management system (CMS) enabling the information about the objects in the book entity. Web semantics are helpful in front-end web applications and also support back-end services. The cultural domain expert provides information about the artwork.

An indoor location-based smart museum has been proposed by Sornalatha et al. [24]. An image recognition algorithm determines objects and wearable devices to capture data from surroundings. BLE-based sensors regulate user location inside the museum. Observed objects are identified from the cloud and narrate to targeted artwork. The cloud helps in streamlining all contents of artwork and provides statistics according to location; the position of the user and different media contents are provided based on age. SNOPS [25] is a smart city project based on future IoT-based multilayered architecture. Multimedia services display information about out-door cultural heritage. SNOPs is useful in what is an artwork’s features? What is the contextual meaning of these information, and how user respond. Many features are defined in the T-Box model. An Android app is used in the case study and verifies different aspects of the proposed smart museum.

Indoor location based on BLE beacons for smart museums has been proposed by Plataniotis et al. [26] to enhance consumer experiences inside museums. The proximity and location services based on Bluetooth low energy (BLE) are provided, which are vital for any smart museum, galleries, and art work. For user movement and detection inside the museum, the user’s location, an app, and a Kalman filter are deployed. Location services, precision, and accuracy of the user inside the museum are delivered by cloud services. The system was theoretically checked in terms of location awareness, detection accuracy, and distance computational accuracy. A multitier framework model suggested by Deebak et al. [27] is another smart context-aware system for museums on the IoT–Cloud (IoT–BSFCAN). Consumer, cloud, and sensor/virtual server are the three layers of the IoT–BSFCAN. For workload balancing, cloud services are employed in two scenarios. Sensor signature, data flow, and allocations are used to analyze the cloud services in smart devices. The model was assessed for better service execution and energy consumption.

Behavior modeling for a beacon-based indoor location system [28], proposed by Bilbao-Jayo et al., is based on smartphones and smart watches for monitoring devices. For indoor navigation, two approaches based on holistic algorithms are used to predict behavior. Analysis and performance evaluations are assisted by several parameters. Neural embedding is used to pinpoint any place within a building or household. Machine learning implements the deep hidden Markova procedure. For the recognition module, the system can also use RFID-based architecture. Another scheme for a smart interactive pattern-recognition system for cultural heritage [29] has been proposed by Balducci et al. The proposed solution can be used to develop innovative immersive content in interactive IoT-based cultural heritage. It incorporates pattern recognition and computer vision techniques to aid CH professionals in the creation of smart interactive experiences by customizing the behavior of the smart objects involved. The results of an experimental examination of the approaches utilized are given and discussed.

López-Martínez et al. proposed a low-maintenance gamified smart object platform that uses semantic web technologies to automate the development of questions [30]. In this scheme, the idea of embedding smart objects in museums, providing exhibitions with advanced interactive capabilities, makes the upcoming Internet of Things (IoT) technology particularly appealing. Gamification tactics can also be used to attract visitors’ attention. These are frequently based on interactive question-and-answer games. One disadvantage of such games is that questions must be regenerated on a regular basis, which is a time-consuming operation. González et al. proposed another scheme, “Cultural Heritage and Internet of Things” [31], for IoT-based cultural heritage. The proposed solution provides a short but representative overview of existing IoT solutions used for cultural heritage cases, both to improve user experience and preventive conservation. Not only are the most intriguing ones emphasized, but also those isolated “exercises” that do not attract effective attention due to lack of applicability or difficulties in reusing for other situations. Finally, the proposed scheme enlist all challenges in IoT-based CH and its impact on real world objects.

Another article provides an overview of the use of sensing technologies in the field of historic preservation [32], with a focus on fiber optic sensing and wireless sensor networks. Ancient structures are an essential element of the world’s cultural legacy and a nonrenewable resource, together with providing historical data for understanding social economics, culture and art, and religious beliefs throughout the period of ancient buildings. However, with society’s fast expansion, particularly the rise of urban construction, pollution, and tourism, the safety of these old objects are more critical. To meet the objective of providing data support for the preventative preservation programmers of ancient structures, a full system with multi-node sensors for the environmental monitoring of ancient buildings is proposed [33]. In this scheme, hardware construction of the monitoring platform and packaging has been designed. This system measures eleven different environmental factors and employs ten different types of sensors. The new indoor node package box and outside package device were designed and built to meet the demands of historic structures. The layered structural design of the indoor node package box may separate the node module from the sensor module, which not only protects the node module from dust and water, but also facilitates replacement in the event of sensor module damage.

Table 1 summarizes all of the proposed solutions for smart museums, which are primarily focused on the presentation of information in a variety forms such as text, audio, multimedia, maps, and web links. Although the information provided in these formats is useful, it does not attract the interest of visitors and receives no charm. In the proposed model, the information is delivered through storytelling. Similarly, the range of choices for presenting information in the previous approaches is quite limited, which not only leads to a lack of interest but also distracts visitors. The SNIS has been designed in such a manner that it captures visitors’ interest and captivates their curiosity.

## 3. Proposed Model of Smart Navigation and Information System

The smart navigation and information system (SNIS) provides an immersive and dynamic framework for understanding the objects available at a museum. The museum’s items incorporate embedded sensors for position awareness together with communication with surrounding objects and visitors’ smartphones.

### 3.1. Museum and Archaeological Sites for SNIS

The Chakdara Museum in Dir Lower, Khyber-Pakhtunkhwa, Pakistan, was chosen for the prototype’s real-world implementation because it is the only local museum having appropriate archaeological and historical artifacts required for the project. Chakdara Museum was founded in 1977 and exhibits 2243 items spanning from 2000 B.C. to 1700 A.D. This museum is rich in cultural items—from Buddha statues to Gandhara art—together with many other things from the past. Different archaeological items, including statues, coins, sculptures, ornaments, decoration pieces, etc., are displayed in the museum. These items were discovered and collected from local archaeological sites near the Chakdara Museum’s premises. The museum has six registered archaeological sites; their names and estimated distance from the museum follow: (1) Chat-Pat (2.5 km), (2) Andan Dheri (7 km), (3) Gumbat Fort (15 km), (4) Timargara Graveyard (30 km), (5) Bambolai (9 km), and (6) Ramora (4 km).

### 3.2. Architecture of SNIS

**Figure 1:**
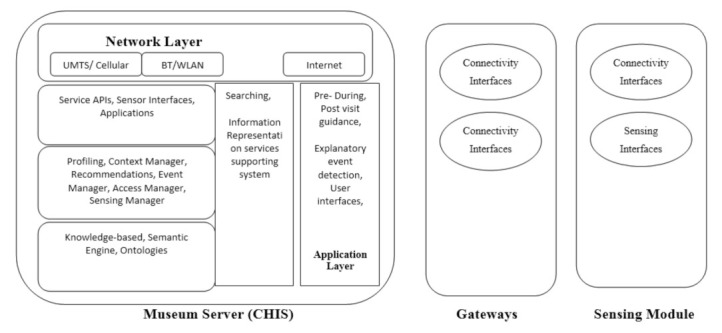
Modified three-layered architecture.

The three-layer architecture for IoT was modified for the deployment of the proposed system [17,34]. Between the background process and front-end application services, this article adds searching, services supporting system, and information representation. Sensor module, network layer, and application layer are all part of the layered architecture. The sensor module collects data from surrounding sensor nodes and visitors. The gateway’s objective is to keep network-level activities, including data formats and communication channels, operating properly. The network layer is responsible for application-level activities, including configuring heterogeneous network architecture and applications. The application layer provides middle-ware services for IoT architecture [17]. The application layer services are added, including pre-visit, during-visit, post-visit guidance, together with explanatory event description and user interface for various activities. The application layer easily searches for a specific cultural item using its extra-services support. The function of the three-layered architecture is shown in Figure 1. It is added as a sensing module, gateways, application layer, and network layer with background functionalities. The application layer and network layer with system features are combined in the museum server (MuServer).

### 3.3. Deployment of SNIS

**Figure 2:**
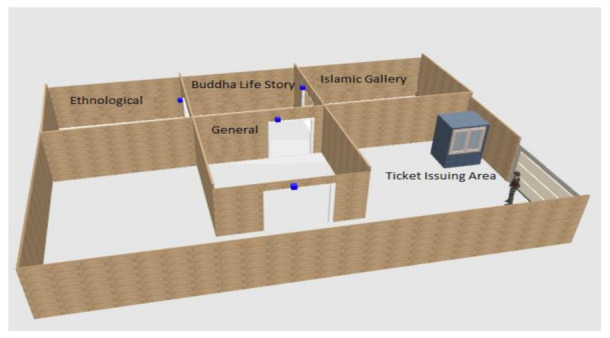
Three-dimensional map of Chakdara Museum.

The Chakdara Museum building is sectioned into main four parts, i.e., General Section, Buddha Life Story, Islamic Gallery, and Ethnological Section. The General section is decorated with different objects including stone statues of a later time of Buddha. Buddha Life Story is also known as the Gandhara Art Gallery, having stone statues depicting different life scenes of Buddha-like Buddha seated in a cave, Buddha leaving a palace scene, the mad elephant and Buddha, musicians, dancers, lovers in rows or drinking scene, etc. Similarly, the Islamic Gallery is also known as the Islamic Art Gallery, having a quality collection of holy books written by hand. Lastly, the Ethnological Section, also known as the “Hall of Tribes”, is adorned with cultural heritage objects including dresses, ornaments, musical instruments, household objects, armory, and religion. A three-dimensional map of the building is shown in Figure 2.

### 3.4. Deployment of the Proposed System

SNIS is deployed as a framework of navigation and information system where a user is guided about all the practices. Each user is given directions on how to start the visit, where to start the visit, and how to proceed within the museum. When visitors gain access to the internet and download the museum application (MuApps), it directs them to the starting point and then suggests to them where to go next based on their preferences. The visitor is guided by the navigation system, which uses arrows pointing in different directions, all pointing to a different item of interest. When a user approaches an object, MuApps displays textual information and an audio message to explore the object. MuApps displays all information about a specific object on the screen and provides links to detailed information.

#### 3.4.1. Deployment of Sensors for Indoor Localization

A set of sensors detects user movement throughout museum. These sensors communicate with the visitor’s smartphone to determine where the visitor moves and stands within the building. The Bluetooth low energy (BLE) chip is available at an affordable power and low energy consumption. BLE is primarily used for managing data transfer rates, data exposure, radio signal transmission, and connection establishment and data transfer delays [35]. BLE is ideal where IoT and sensor technologies are in emergence with client–server architecture. The BLE–BlueNRG-MS network processor is uniquely identified by public addressing and random addressing modes. Random addressing is handled in static manner depends upon the real time scenario. The BlueNRG-MS stack runs on an embedded Cortex M0 core with core specification version 4.1 [36]. The BLE components are marked with a unique address mechanism and known as museum sensors (MuSens). BLE is better approach due its good qualities of low power consumption, ad hoc connectivity, less complexity, and better security. It is used in WSNs for data collection and real-time sensation in the environments. Many schemes have used it for indoor monitoring systems that are designed to monitor building interiors. Environmental parameters such as temperature and humidity are quantified. These values are stable, easy to use, speedy, reliable, and flexible [37,38].

MuSens are deployed on these cultural objects directly or are installed very near the object. MuSens collect data from these objects and also from its surroundings and send to MuServer for further analysis. Every BLE MuSens collect data on the number of users around its premises and its specific distance from certain cultural objects (Obij) by using the subsequent value of the receive signal strength indicator (RSSI). The equation for calculating RSSI value is RSSI = (10n log10 d + A), in which “d” is the distance of the user from Obij, “A” is the value of the signal strength received at one meter, and “n” is a constant used for signal propagation [39]. The RSSI value is the power for hearing another device. RSSI values are measured in decibels from 0 to −120. The value closer to one indicates a stronger signal [40]. The values determine the location of the user within the premises of a specific object. Depending on the value, the user location is specified and content is provided based on its location. MuSens implements a proximity algorithm and RSSI values to determine the surroundings for neighboring nodes and visitors standing close to these objects. This reflects the overall image of the building along with the visitor’s position. Every object in the museum has a BLE–BlueNRG-MS and computed its premises for smartphone detection. If the strength of the signal (RSSI) is equal between two objects (RSSIA = RSSIB), then an AutoMsgi (“Move Closer your Phone towards Object Please”) will be displayed on the screen. When the RSSI value changes according to the object, the contents will change. RSSI is primarily used to calculate the distance between two objects. The accuracy and precision of indoor localization are determined by object detection and accurate distance. Algorithm 1 calculates the distance between objects using the basic parameter of RSSI value.

**Algorithm** **1** Calculate RSSI-based Distance[*Obj ij, RSSIi, RSSIj, Threshold, dB* ≥ *∨ dB* ≠ 0]High intensity Obj (ij) i.e.dBi ≥ dBj1: Search for availability of RSSI Compared the values of all available RSSI    if (*RSSIA ≥ RSSIB*) then    select RSSIA2: For data gathering and established connection with MuServer.    if *RSSIA == RSSIB* then    Display Message (Move closer to an Object Please)    Concatenate all services related to RSSIA from MuServer.3: Repeat 1–24: End

**Figure 3:**
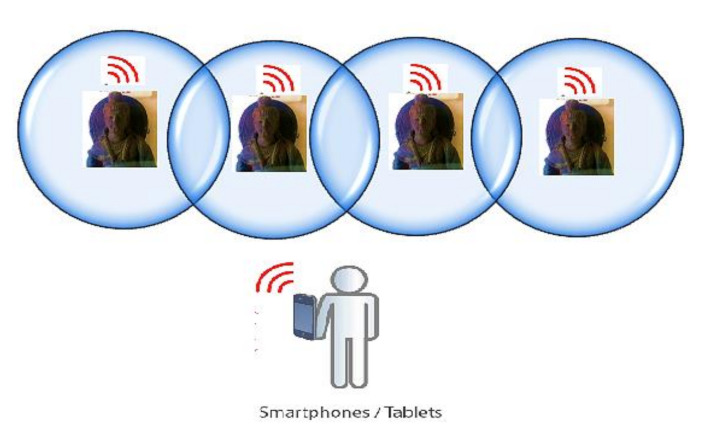
Sensor premises around an object.

The RSSI is important in determining the visitor’s location, relative distance from the other objects, and user direction inside a museum. MuSense collaborates to create and combines content about objects and visitors. When a visitor enters the museum and starts his or her visit with a specific object, MuApps navigates back to the starting position and continues through the building. Depending on the choice, MuApps navigates to specific objects and streams information to the visitor’s smartphone/tablet. In Figure 3, the premises of each object are shown. When a visitor comes near and scrolls the smartphone camera toward an object, then the MuSense (BLE) detects the user movement and a torrent of information is received from MuServer.

**Figure 4:**
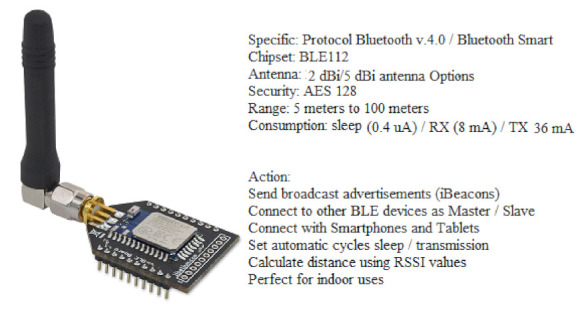
BLE/MuSense used in SNIS [22].

In Figure 4, BLE use in the prototype is shown with its specifications and actions [41]. This device is used in many applications and projects due to its easy deployment and low cost [42]. This component is attached directly or fastened to cultural objects and creates a premise and location around each object. Once the visitor having a smartphone/tablet comes near, the object will detect the device and provides information according to the context and its location. In Algorithm (2), finding a BLE device and establishing its connection is shown.

**Algorithm** **2** Searching BLE devices[*Obj_ij_, RSSI_i_, RSSI_j_, Threshold, dB* ≥ ∨ *dB* ≠ 0]
*High intensity Obj (ij) i.e.dBi ≥ dBj*
1: Initializing Obj_ij_ for searching spaces2: Repeat (1: n) for all *Obj_ij_ DO*3: Calculate High intensity Obj_ij_    *IF BLE (Obj) > for all Obj_ij_*      *Obj_ij_* is main device      *Else-if (check all Obj_ij_)*    *Select Obj (n)*    *End-if*

#### 3.4.2. Deployment of Network

**Figure 5:**
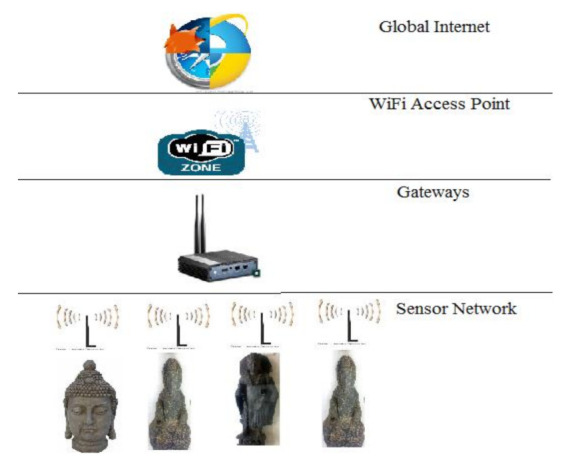
Network layer.

For implementation of SNIS, a WiFi network is deployed throughout the museum building. MuServer maintains a list of visitors currently inside the museum and also determines their locations. A user name and password are provided for connecting with the system and also for using services provided by MuServer. MuSens/BLE collects data from its surrounding and sends it to gateways for WiFi access to MuServer. Visitors use this network to search for other related links and for sharing facility features if the visitor wants to access social media. At the basic level, communication between the cultural object and the visitor’s device is implemented by MuSens/BLE. Data are collected to gateways from these components for access to the WiFi network with MuServer and then using global internet for accessing social media and other links. Deployment of the network layer is shown in Figure 5 in which the working procedure and hierarchy of the different network architecture and integration have been shown.

#### 3.4.3. Smartphone App

MuApps provides user interfaces to connect with the system and provides information in an understandable and convincing manner. The MuApps is an easy-to-use service layer and the visitor scrolls his/her smartphone camera CCi toward an object Objij. MuApps reads it and through the proximity algorithm with RSSI value, detects its current position. MuServer streams information to the front-end application in various forms. MuApps provides three types of lists: 1) list of options for selecting a specific section in the museum, 2) list of options for selecting user profile, 3) list of options for selecting the type of story/choice. After selecting each section, MuApps navigates the visitor to the appropriate section and cascades suitable contents according to a user profile. Story selection navigates the visitor to proper objects and different statues in which the story is revealed.

#### 3.4.4. RSSI-Based Location Accuracy

**Figure 6:**
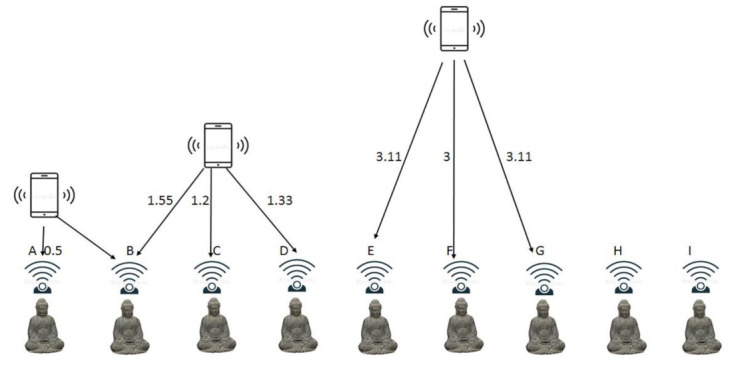
Determining the location and distances with three different points.

For accuracy and reliability of visitor’s location inside museum, “10n log10 d + A” is used. Different sensors communicate each other and with the visitor’s smartphone. In SNIS, different statues (MuSens) communicate with smartphone sensors and provide location and other services (storytelling). In receiving mode, RSSI values are continuously read by the RSSI status register in binary complement number. For decision, these binary values are converted to absolute power value. The accuracy of the visitor’s location is calculated from these RSSI values. The location and RSSI values are calculated and analyzed at different locations. The distance and different values of RSSI are shown in Table 2. For accuracy, six objects (Obj_A_, Obj_B_, Obj_C_, Obj_D_, Obj_E_, Obj_F_) are placed in two meters apart and 0.333 m from each other, as shown in Figure 6. The visitor’s location (smartphone) is placed at three different positions and calculates the distance with RSSI values. In the first case, the visitor’s location is 0.5 m away from Obj_A_ and other statue distances are measured and noted in the table. The respective values of RSSI from Obj_A_ to Obj_F_ are quantified and noted in the table. The MuServer provides services (location and other related information) concerning the statue/object that indicates the maximum RSSI value. In this case, Obj_A_ is nearest to the visitor’s phone and information is synchronized to the same device. In the second case, the visitor is standing at a distance of 1.20 m from Obj_C_. Based on the RSSI values, MuServer provides information to visitor about Obj_C_ because the RSSI value is maximum. When the visitor stands at distance of 3 m from Obj_E_, corresponding distances and RSSI values are determined. Given the high value, services are provided to Obj_E_ and then switched if moved in any direction.

#### 3.4.5. Interference between BLE and WiFi in SNIS

There is a WiFi network inside museum with each Obj_N_ fastened to a BLE for better connectivity and lower energy consumption. These technologies occasionally interfere with each other because the same frequency band is used [43,44]. There are multiple objects (Obj_N_) inside the museum and each visitor has his/her own smart device to visit/enjoy the contents inside museum. There is fluctuation in the signal strength of WiFi while BLE have minimum range with little distortion. For interference, the environment was tested by analyzing different values of RSSI for both WiFi and BLE. From these values, a packet delivery was calculated and quantified, as shown in Table 3. However, few experiments were conducted and few results obtained; therefore, further experimentation is needed.

### 3.5. System Model

**Figure 7:**
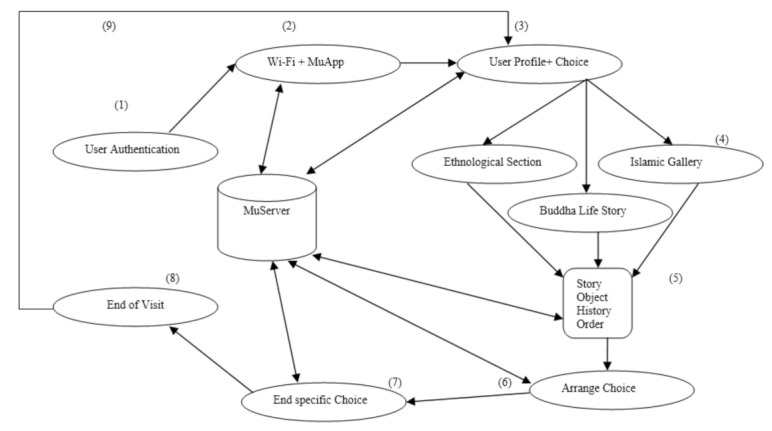
System level view of SNIS.

In the background, MuApps collaborates with MuServer in nine distinct steps. In Figure 7, the system-level view of the navigation system is shown. This section explains the SNIS guidance and navigation of a visitor from the start to the end of his/her tour, and explains each step of the proposed model in detail.

Step 1. MuApps is installed on visitors’ smartphones. A username and password are provided for accessing the services.

Step 2. MuServer keeps a list of users who have been authenticated using their username and password.

Step 3. Based on the visitor’s profile and choices, the visitor is properly guided to a specific section inside the museum.

Step 4. MuApps directs the visitor to the appropriate section and needs to announce AutoMsgi, which instructs the visitor on how to start and where to go next.

Step 5. MuApps directs the visitor to the correct section and must announce AutoMsgi, which instructs the visitor on how to start and where to go next.

Step 6. MuServer arranges contents according to user profiles and choices.

Step 7. At the end of the story, the visitor is guided and stimulated to visit additional objects or sections, and shares his or her experience on social media.

Step 8. MuServer announces a vocal message AutoMsgi at end of the visit and suggests further directions.

Step 9. The system navigates the visitor from where to restart and go for the next step.

#### 3.5.1. User Authentication

**Figure 8:**
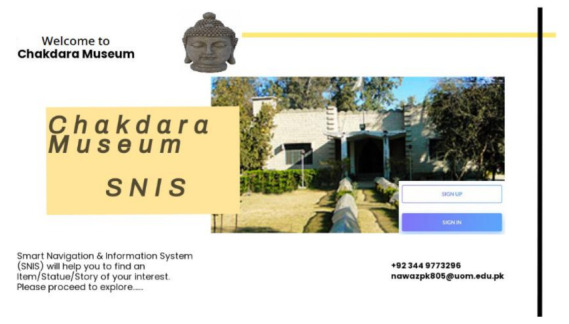
Visitor authentication.

**Figure 9:**
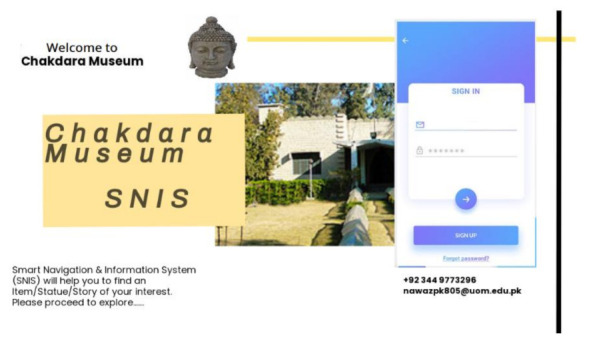
Authentication after receiving a ticket at the help desk.

MuServer authenticates the user by requesting a username and password. The MuServer currently visiting the museum keeps a list of users. The MuApps are installed on the visitors’ smartphones or tablets. The services are provided in accordance with the user’s level of knowledge. Only authentic visitors are provided by MuServer. The user first enters the museum; the ticket is provided by the ticket help desk/ticket issuing. The ticket includes access to the network and installation of MuApps on visitors’ smartphones. When a user is connected to a network, MuServer creates a session for the current user. The user is added to the user list in MuServer user list. This user list helps to determine the number of users in a museum. The user is now a registered user for the current session. User authentication in the MuApps is illustrated in Figure 8 and Figure 9.

Vi → WiFi code + MuApps at Help Disk(1)

#### 3.5.2. User Profiling

**Figure 10:**
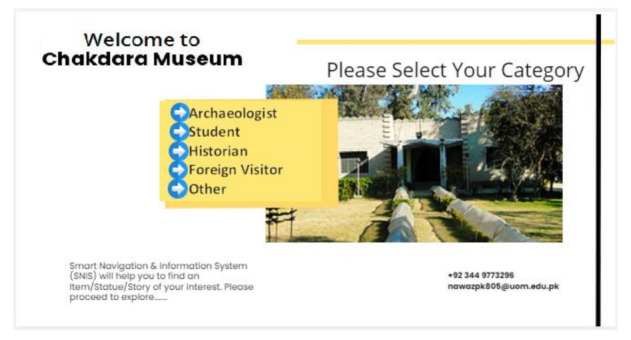
User profile selection.

MuApps offers information about the objects in a museum according to user preferences. MuApps provides a list of choices to select an option according to the interest. The MuServer then presents information based on user choices. Compared to an archaeological expert, the information provided to students is different in presentation and content. The system categorizes the visitors as students, archaeologists, families, foreign visitors, local visitors, and others. When a user selects a category, MuApps provides the information in different forms. The archaeologist needs more in-depth knowledge while a student only visits for pleasure or basic information. Services and multimedia contents are provided according to the selected profile. Figure 10 shows how different users are categorized.

When Vi enters into the main building, an AutoMsgi from the MuServer is displayed on the screen or an audio message is received, “Welcome to Chakdara Museum”. When Vi clicks on the “start button” to begin the visit, MuApps navigates and guides visitors. After clicking the button, a list of options for user type displays on the visitor screen. After selecting a specific type of visitor (archaeologist, student, technical person, historian, etc.) from the list, MuApps streams the information according to his/her knowledge level. Another list will appear on the screen for Vi to select the appropriate section for the visit.

MuServer → AutoMsgi + List of Categories Vi selects the option from the predefined list.(2)

#### 3.5.3. Selection of Story

**Figure 11:**
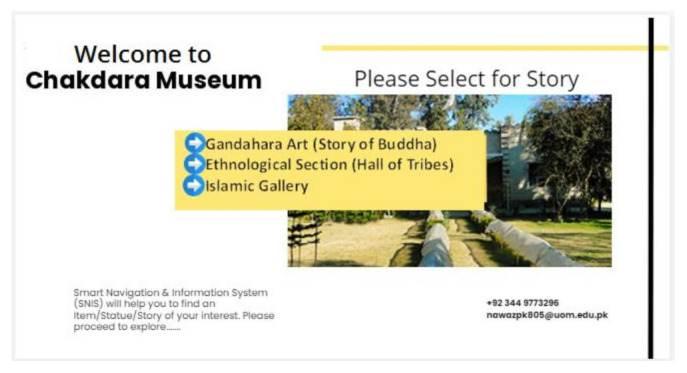
Selecting a section.

**Figure 12:**
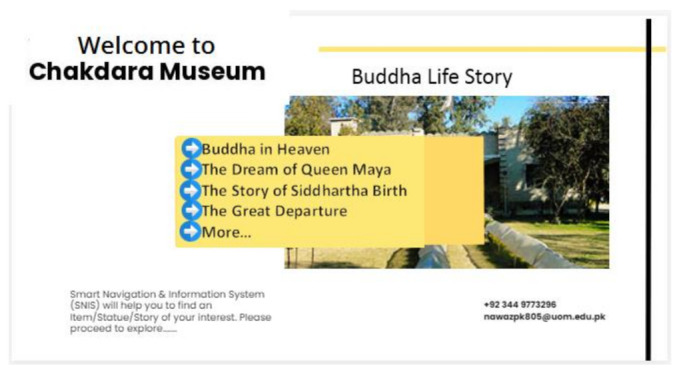
Select a story.

After visitor profiling, three options display on the screen: Gandahara Art (Buddha Life Story), Ethnological Section (Hall of Tribes), and Islamic Gallery. When a user selects one of these three distinct sections, the system navigates to a specific hall/area. The selection of specific section is shown in Figure 11. After selecting a specific section, MuApps provides a list of stories about that section. For example, if a visitor selects Gandahara Art, then the list of stories is displayed on the screen, as shown in Figure 12. We can express it as:

MuServer → AutoMsgi + List of Options(3)

#### 3.5.4. User Navigation

**Figure 13:**
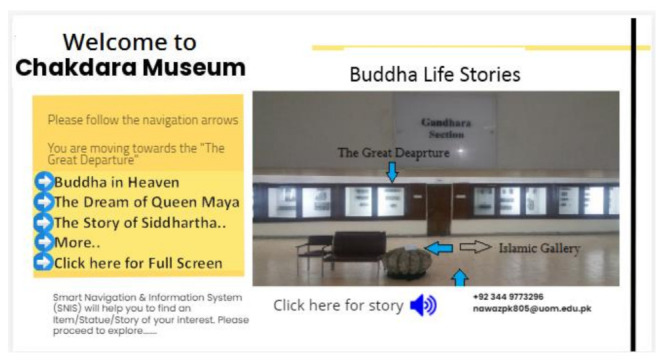
Navigation toward the proper item/location.

**Figure 14:**
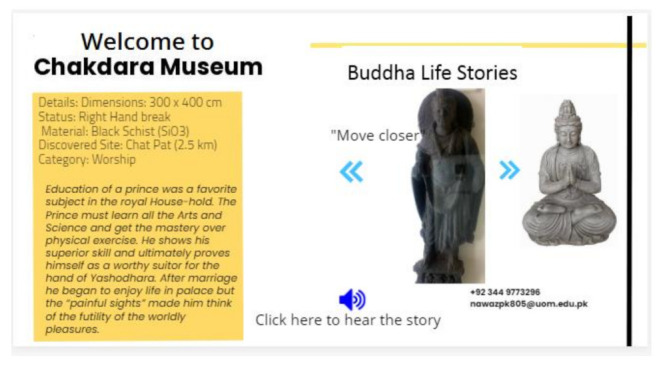
Screen image of the information displayed on the screen.

MuApps navigates visitors according to the structure of the building. After installing MuApps at the ticket issuing section, navigation is started. First, the visitor is navigated around the building and about different sections in the building. After entering into a specific section, the system navigates visitors to the objects and statues in that section. The navigation also connects related items in proper sequence according to the user-selected option for a story or history. In Figure 13, MuApps shows how to navigate to proper sections and objects; Figure 14 shows the screen image of the proper sections and objects.

#### 3.5.5. Information System

The information of all objects is stored in MuServer; MuApps obtains all the pertinent details from MuServer. MuSens are attached to each object in the museum and these sensors detect their neighbors, number of visitors, and user presence by using a proximity algorithm. MuSens collects data about its neighbors and the user and sends it to the gateway for MuServer. MuServer analyzes the position of the user and streams the information according to his/her current standing position. All information about the objects is stored in memory on MuServer. In Figure 14, a MuApps screen image shows the user how to read or play a story about the specific object visible in visitor’s phone camera.

#### 3.5.6. Remote Sites Navigation

**Figure 15:**
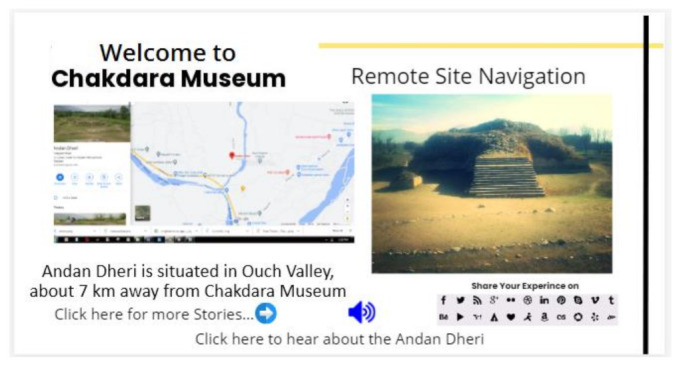
Navigation towards an archeological site.

The system also navigates the user around the registered archaeological sites in the area. When a user clicks for further details and clicks on the site, then the system navigates to the proper site and shows pictures. The user is guided if he/she is interested in visiting this particular site. The navigation tells the visitor the distance in kilometers and direction to the site. The image of the site and Google map image are displayed on the screen. Navigation to archaeological site is shown in Figure 15.

## 4. Experimental Results and System Analysis

### 4.1. User Preferences, Usefulness, and Satisfaction

**Figure 16:**
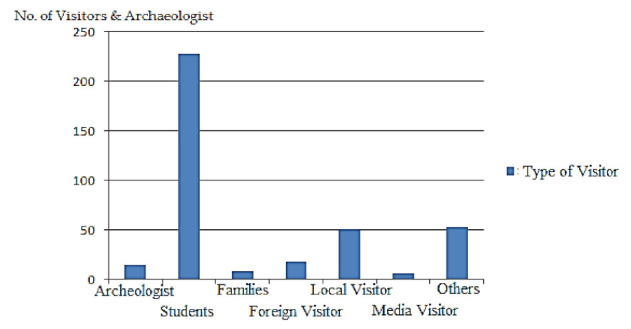
Types and numbers of visitors.

The museum receives a diverse range of visitors. At the end of the visit, MuApps displays a form on which a visitor can provide feedback on the system. The form’s response reflects how users feel about the system and services. For two months, we experimented with MuApps at the Chakdara Museum’s main hall. MuApps is downloaded to every visitor’s smartphone and instructs them on how to use it. In the hall, the visitor uses the smart navigation system to find appropriate sections and specific objects. MuApps represent the overall system with star ratings ranging from top (five star) to lowest (one star), with five-star representing good performance and other stars indicating from better to poorer performance. We also used a percentage of the visitors who have visited in the last two months in Table 4. The visitor categories are marked with stars, and the average choices in the last column are marked with a generalized form of stars. The majority of visitors are students, as indicated by these statistics, yet their average star rating is three with 59.84 percent of all visitors and an average choice of 0.6. The archaeologist, foreign visitors, local visitors, and media visitors all gave the proposed prototype five stars out of a possible one star on a scale of one to five. The maximum number of visitors is categorized into two months of museum visits, as shown in Figure 16. The student’s ratio is more than other categories because most students came to the museum in groups from a school or college. Here, visitor’s preferences (favorites) and choices are distinctive (likes) for each visitor. For each visitor, two parameters have used, preference and choices. Both these values have used in calculating the overall impact of the system. The preferences are ranked from one to five stars, indicating that each visitor assigned a number from one to five stars to each category based on his/her interests. The better the system he/she marks, the higher the score. To determine out how well SNIS works, we take the average of these marking values. Similarly, we quantify the choices, which range from 0.1 to 1.0; the higher the choice, the higher the score. The values range from 0.1 to 1.0, and the average from these values is designated as the average choice.

Visitors can use the feedback form’s six features to analyze the degree of these points and categories into one of five categories. These aspects/qualities are: the system is interesting (1–5), realistic (1–5), easy to use (1–5), service satisfactory (1–5), useful (1–5), and user friendly (1–5). The form has five options: most (highest/excellent), marked as one (1), the first-class category; more (higher/better) category is marked as two (2), the second class category; and so on, through category 5, marked as “do not know”. Statistics of these observations are shown in Table 5. For a total of 381 visitors, “interesting” was marked 201 times, “better” was marked 87 times, “average” was marked 50 times, “system is not interesting” was marked 41 times, and “I do not know” was marked 2 times. In the same way, all the features are presented 381 times while different visitors marked every feature differently. Details of these scores from excellent (1) to bad/do not know (4/5) are shown in Table 5. Three hundred eighty-one individuals visited the museum during the time period of the experiment (2 months). These 381 visitors were evaluated by the system based on various of characteristics, that is, how visitors feel about the system, which can be classified as excellent, better/very good, good, and bad service, or have no knowledge of the system (do not know).

**Figure 17:**
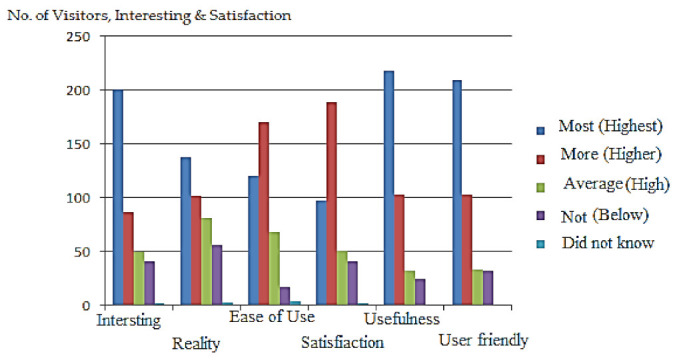
SNIS performance concerning six features and the number of visitors.

Overall system performance is checked and indicated in the graph shown in Figure 17. The number of visitors is plotted on the *y*-axis while features are plotted on the *x*-axis, which shows their comparison. The graph indicates that the majority of visitors chose option one (1) for excellent service, while only a minority chose option two (2) for poor service or did not know about the system’s performance. These findings demonstrate the SNIS’s superior behavior and performance.

### 4.2. System Performance with Increasing Number of Visitors and Objects

Performance of SNIS is analyzed with increasing number of visitors together with an increasing number of objects (statues). With the permission of museum authorities, the “Hall of Tribes” was adorned with a changing number of objects for experimentation. Other museum sections were fixed with statues and cannot be changed, but the “Hall of Tribes” can be altered and objects added or removed. We added some objects and used MuSens to embed them. The results were obtained from deployed objects in various scenarios and analyzed in different aspects.

**Figure 18:**
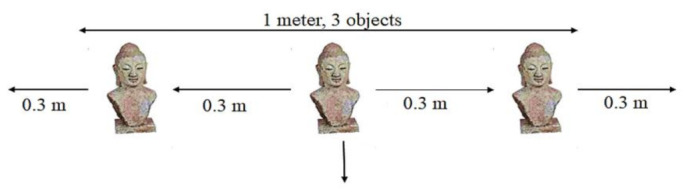
Three objects are placed within one meter.

**Figure 19:**
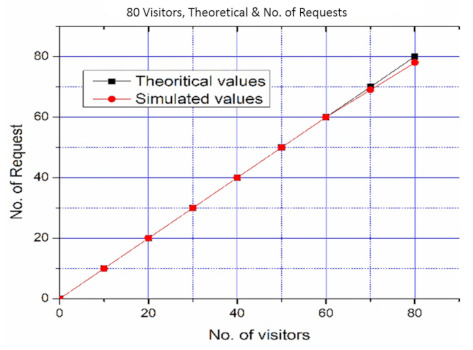
SNIS Performance with 480 Objects and 80 Visitors.

First, the Hall of Tribes (50 × 30 feet) was adorned with 480 objects, each measuring three meters in length. As shown in Figure 18, all objects are roughly 0.3 m apart around the hall near the wall-sides. This scenario was trialed with 80 visitors at one time and all these visitors were engaged to request and obtained the position of different objects. With a rising number of visitors, the scenario was performed. First, ten visitors were permitted, each of whom was assigned to a different section of the hall. All of these visitors were pleased with the services provided. Afterward, a total of ten additional visitors were allowed to use the system. These 20 visitors all indicated better results, and they received them by checking services in various parts of the hall. Finally, 70 people were permitted to enter the hall. They tried the SNIS and provided the same results. Because SNIS provides services in the form of stories, the system responds to all demands for services in a timely and error-free style. These results are consistent with theoretical values. It means that with 70 visitors, SNIS performed consistently. When 80 people are allowed to access the services when there are 480 objects in the same hall, the system performance degrades, as illustrated in Figure 19. This is due to the fact that the “Hall of Tribes” is 50 × 30 feet in size, and these statues are positioned near the walls, serving all of these visitors at once, affecting system performance. The size of the hall is insufficient to accommodate all of these visitors; the objects are fastened to the walls, thus causing congestion and degrading system performance.

**Figure 20:**
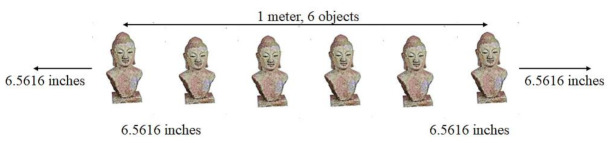
Doubling the number of objects within the same area (1 m, 6 objects).

**Figure 21:**
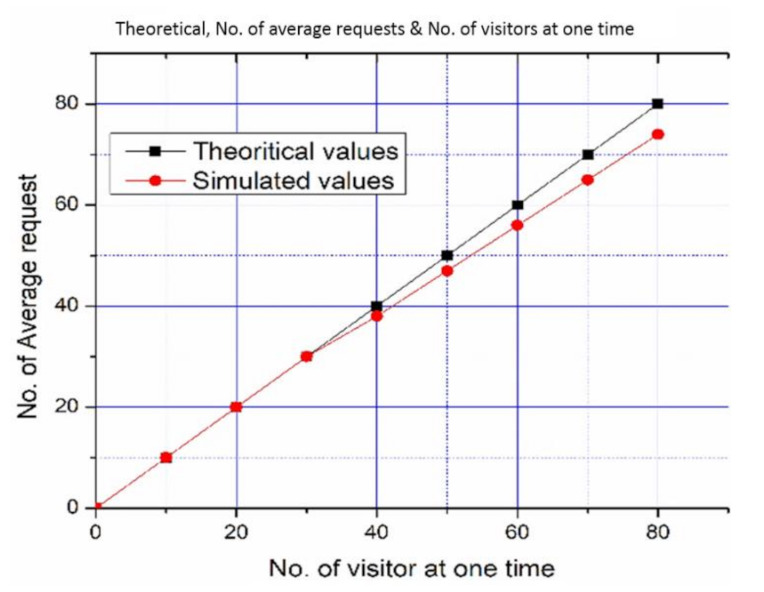
SNIS performance with 960 Objects and 80 Visitors.

SNIS behavior was also analyzed with increasing statues and the same number of visitors (80). The statues along the walls were adorned in a congested manner, as shown Figure 20. Now, there is a clear difference between theoretical and actual results, as shown in Figure 21. The experiment started with increasing number of visitors. The system performed well with up to 30 visitors, but the system showed diverging results from theoretical values with increasing number of visitors.

The variations in the results have been shown in Figure 21. SNIS in 960 Objects, 80 Visitors.

Table 6 with percent change and error ratio. After 30 visitors, SNIS showed no variation, but deviation started with a larger number of visitors. The standard deviation is the variation in findings that SNIS displays when compared to its mean position. The results show that when the number of visitors to the museum increases, the variance grows as well. The variance is increases because of increasing number of visitors inside museum. It is due to an increase in the number of visitors to the museum and a corresponding increase in the demand for SNIS services. The standard error is still less than one, indicating that the error rate is still manageable and can be ignored or discarded.

## 5. Conclusions and Future Work

With the proliferation of embedded systems and technological advancement, the embedding of electronics into everyday physical objects that pervasively and ubiquitously interact with each other results in a connected network known as IoT. The lifeless and dreary objects inside museums, exhibitions, and cultural sites become intelligent and convincing using IoT tools and services. The use of IoT-enabled technology to create smart cultural heritage is becoming more popular. Museum exploration can be made more exciting and informative by deploying IoT technology-based information systems. A new technique of distributing information in the form of stories is presented as a solution to the problem. These stories are told from inside the museum, and the system directs visitors to the appropriate objects depending on the stories. In this way, visitors not only receive information about the objects, but also feel pleasure when experiencing such sequencing of artwork. The story-based information is more stimulating for visitors compared to text or audio information. The proposed prototype demonstrates the assistance, guidance, and navigation within the museum, and explores the information concerning the visitor’s interest and desire. However, by increasing the number of visitors and objects, the performance of the system decreases. The system can be improved in the future by offering state-of-the-art hardware, a museum larger than Chakdara to minimize congestion, and a demonstration to visitors about how to operate SNIS.

## Figures and Tables

**Table 1:** Summary of literature.

Scheme	Strengths	Data Acquisition Techniques	Parameters	Demerits
Alletto et al.	Clear image acquisition	Wearable Device	RSSI, Blurriness formula	Costly, extra devices needed
Chianese et al. (CHIS)	User and semantic-based	Sensors and Cameras	Knowledge Base, Ontology	Focused on user behavior
Chianese et al. (S3)	Museum	Sensors	Proximity, Smart cricket	More technical issues
Haugstvedt et al.	Better multimedia services	Camera	TEAM/List view	Degrade Qos
Chianese et al. (TM)	Multimedia services	Bluetooth	MAC, Gateways	Costly resources
Chianese et al. (MV)	Voice and better image acquisition	Sensors	Master/Slave sensor, CHIS	Needs synchronization
Angelaccio et al. (SV)	Improve delay and image algorithm	Sensors	NFC, Token	Limited range
Chianese et al. (SOA)	Good multimedia	Sensors	CMS, Booklet	Complex structure
Sornalatha et al.	Cloud-based architecture	BLE Sensors	Cloud Services	Needs cloud
Flora Amato et al.	Improved and better services	Sensors	T-Box, 3-layered	Very complex
Plataniotis et al.	Cloud and server based	BLE Sensors	Cloud services	Complex
Deebak et al.	Minimum blurred effect	Sensors	Cloud Service	Costly
Bilbao-Jayo et al.	Navigation system	BLE Sensors	RFIDs	Navigation problems
Balducci et al.	Better multimedia services	Sensors	Proximity	Complex
López-Martínez et al.	Semantic-based services	Sensors	Semantic web	Time consuming

**Table 2:** Distance (meters) and location (decibels).

Test Cases	Parameter	Obj_A_	Obj_B_	Obj_C_	Obj_D_	Obj_E_	Obj_F_
Vi standing near Obj_A_ at a distance of 0.5 m	Distance (meters)	0.5	0.8333	1.1666	1.499	1.832	2.165
	RSSI (decibel)	−59.0	−68.0	−71.72	−74.21	−77.33	−79.34
Vi standing near Obj_B_ at a distance of 1.20 m	Distance (meters)	1.88	1.55	1.20	1.3332	1.444	1.0005
	RSSI (decibel)	−78.8	−75.0	−72.1	−73.2	−73.99	−49.11
Vi standing near Obj_E_ at a distance of 3.0 m	Distance (meters)	4.778	3.998	3. 443	3.11	3	3.11
	RSSI (decibel)	−97.99	−95.78	−91.33	−89.8	−87.33	−89.7

**Table 3:** Interference of WiFi and BLE.

Timer	Data Rate of WiFi	Total Packets Transmitted by WiFi	Total Packets Received by WiFi	Data Rate of BLE	Total Packets Transmitted by BLE	Total Packets Received by BLE	Interference of WiFi
T1	108 kbps	2050	2050	0 Mbps	0	0	0.00%
T2	108 kbps	2079	1921	1 Mbps	2072	2011	7.599%
T3	540 kbps	1918	1849	1 Mbps	2019	2003	3.597%
T4	1080 kbps	2213	2188	1 Mbps	2189	2157	1.129%
T5	2040 kbps	2163	2033	1 Mbps	2089	2056	6.010%

**Table 4:** Types of visitors and preference.

Type of Visitor	No. of Visits	Average Preferences	Percentage	Average Choice
Archaeologist	15	5 stars	3.937	1.0
Students	228	3 stars	59.84	0.6
Families	09	2 stars	2.362	0.4
Foreign Visitor	18	5 stars	4.724	1.0
Local Visitor	51	5 stars	13.385	1.0
Media Visitor	07	5 stars	1.837	1.0
Others	53	1 star	13.910	0.2
Total	381	-	-	-

**Table 5:** Statistics collected from different visitors.

Visitor Experience	Most (Excellent)	More (Better)	Average (Good)	Not (Bad)	Do not know
Interesting	201	87	50	41	2
Reality	138	102	81	57	3
Ease of Use	121	171	68	17	4
Satisfaction	98	189	51	41	2
Usefulness	219	103	33	25	1
User Friendly	210	103	34	33	1

**Table 6:** Types of users by references.

No. of Visitors	No. of Requests Served	Theoretical Values	Simulated Values	Percentage Change	Standard Deviation	Standard Error
10	10	10	10	0.0	0.0	0.0
20	20	20	20	0.0	0.0	0.0
30	30	30	30	0.0	0.0	0.0
40	39	40	39	2.5641	1.4142	0.3162
50	48	50	48	4.1666	2.1213	0.4743
60	57	60	57	5.2631	2.8284	0.6324
70	66	70	66	6.060	3.5355	0.7905
80	75	80	75	6.666	4.2426	0.9486
